# Case report of EUS-guided endoscopic transduodenal necrosectomy in a patient with sleeve gastrectomy

**DOI:** 10.1186/s40608-016-0119-z

**Published:** 2016-09-13

**Authors:** Avik Sarkar, Ragui Sadek, Matthew Lissauer, Swati Pawa

**Affiliations:** 1 Division of Gastroenterology, Department of Internal Medicine, Rutgers - Robert Wood Johnson Medical School, 1 Robert Wood Johnson Place, Medical Education Building Room 478, New Brunswick, NJ 08901 USA; 2 Department of Surgery, Rutgers - Robert Wood Johnson Medical School, New Brunswick, NJ USA

**Keywords:** Sleeve gastrectomy endoscopic necrosectomy, Altered gastric anatomy endoscopic necrosectomy

## Abstract

**Background:**

After an acute attack of pancreatitis, walled-off pancreatic fluid collections (PFC) occur in approximately 10 % of cases. Drainage of the cavity is recommended when specific indications are met. Endoscopic drainage has been adopted as the main intervention for symptomatic walled-off PFC. Altered gastric anatomy in these patients poses an interesting challenge. We present the first case of a patient with sleeve gastrectomy who underwent successful endoscopic transduodenal necrosectomy (TDN).

**Case presentation:**

Forty year old woman with history of morbid obesity status post sleeve gastrectomy in 2009 was found to have symptomatic gallstone disease complicated by severe necrotizing gallstone pancreatitis and further complicated by symptomatic walled off pancreatic necrosis (WOPN). Imaging significant for 10.8 × 7.6 cm fluid collection with necrotic debris in the body and tail of the pancreas and endoscopic necrosectomy was attempted. EGD showed tubular gastric body and antrum, with extrinsic compression in the antrum and duodenal bulb from the pancreatic cyst. Duodenal bulb was selected as the preferred fistula site due to sleeve gastrectomy. Patient underwent successful TDN in two sessions. Patient had symptomatic improvement at follow-up with resolution of WOPN.

**Conclusion:**

To our knowledge, this is the first reported case of EUS-guided endoscopic necrosectomy in a patient with sleeve gastrectomy. The duodenal approach was used in our patient due to history of sleeve gastrectomy.

## Background

After an attack of severe acute pancreatitis, walled-off pancreatic fluid or necrotic collections occur in nearly 10 % of cases [1]. Decompression is recommended in symptomatic patients with abdominal pain, gastric outlet or biliary obstruction, fluid leakage, fistulization, weight loss or failure to thrive, and infection [2, 3]. Controversy exists regarding initial therapy but endoscopic ultrasound (EUS) guided transmural drainage has been adopted as the first line therapy for symptomatic pseudocysts and walled off pancreatic necrosis (WOPN) given its similar efficacy, shorter recovery times, low rate of adverse events and improved cost-effectiveness compared with surgical cystgastrostomy [2, 4–8]. Endoscopic treatment outcomes are directly related to the type of pancreatic fluid collection (PFC) being treated; while the treatment success for pancreatic pseudocysts is greater than 90 %, it is 50 to 65 % for WOPN [9, 10]. With this method, the puncture route with the shortest distance between the gastrointestinal tract and the wall of the cyst is chosen under EUS guidance, and most often this is a transgastric approach. A large bore needle is used to access the identified pseudocyst or WOPN, creating a fistula between the cystic cavity and the gastrointestinal tract [11]. In cases of altered gastric anatomy, there may be technical difficulty due to limited space and patients may require surgical or laparoscopic cystgastrostomy [12]. We present an index case of a patient with sleeve gastrectomy who underwent successful endoscopic transduodenal necrosectomy (TDN).

## Case presentation

**Fig. 1:**
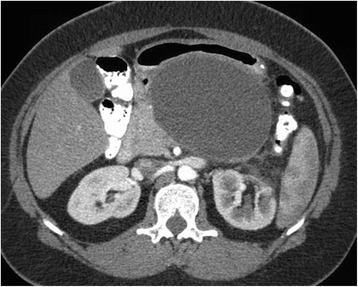
Duodenal compression (axial CT image)

**Fig. 2:**
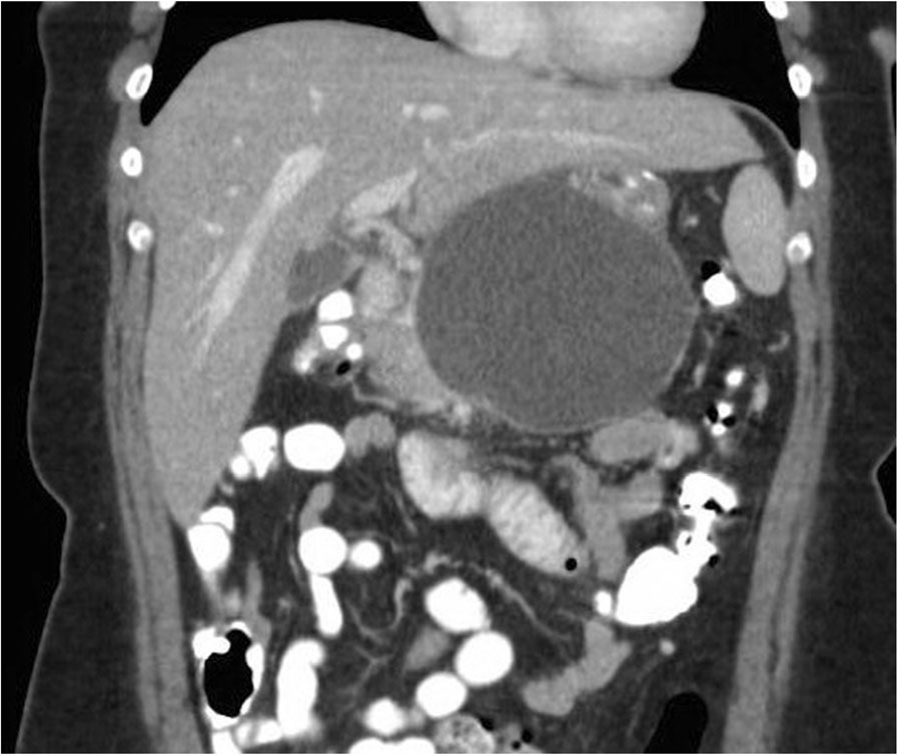
WOPN imaging (coronal CT image)

**Fig. 3:**
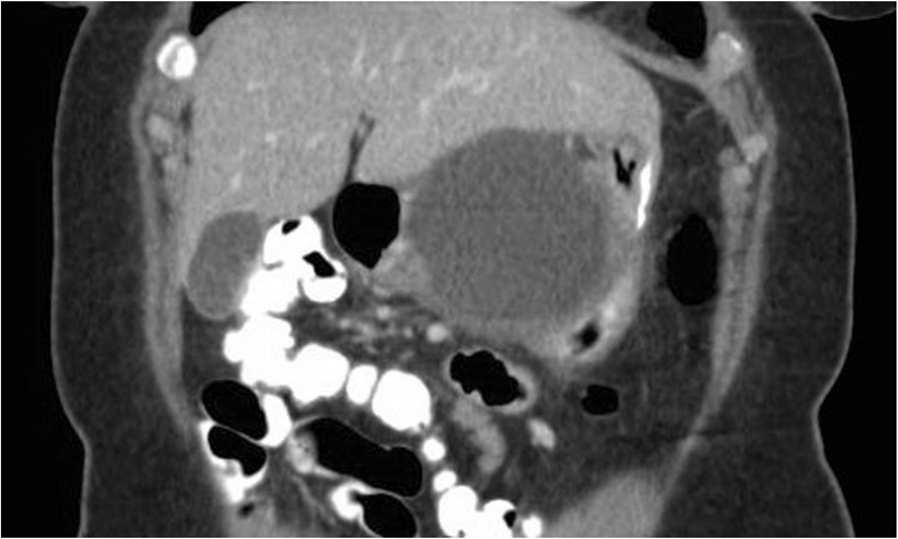
Sleeve compression (coronal CT image)

**Fig. 4:**
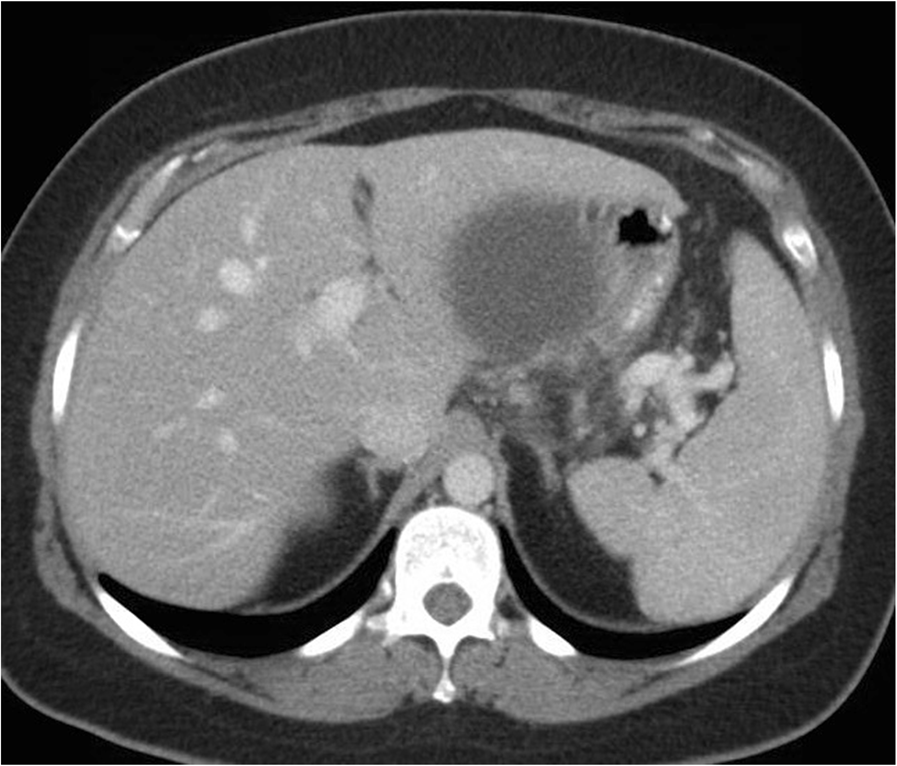
Sleeve compression (axial CT image)

**Fig. 5:**
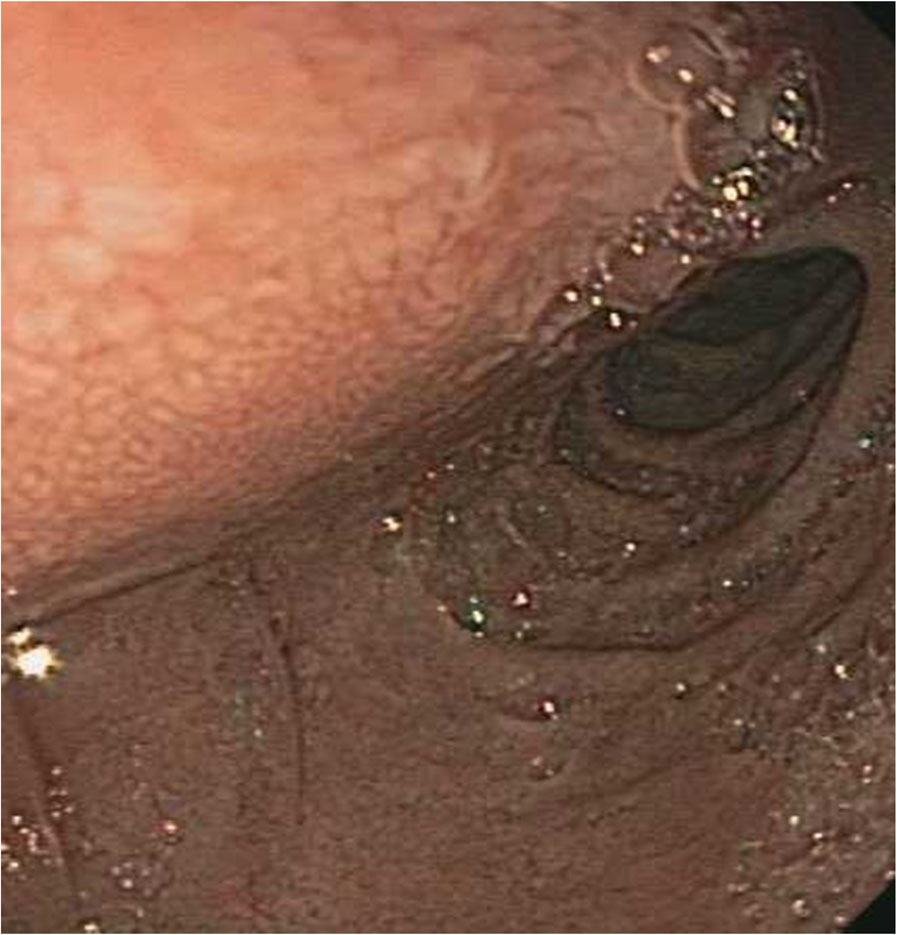
Duodenal bulb extrinsic compression from WOPN

**Fig. 6:**
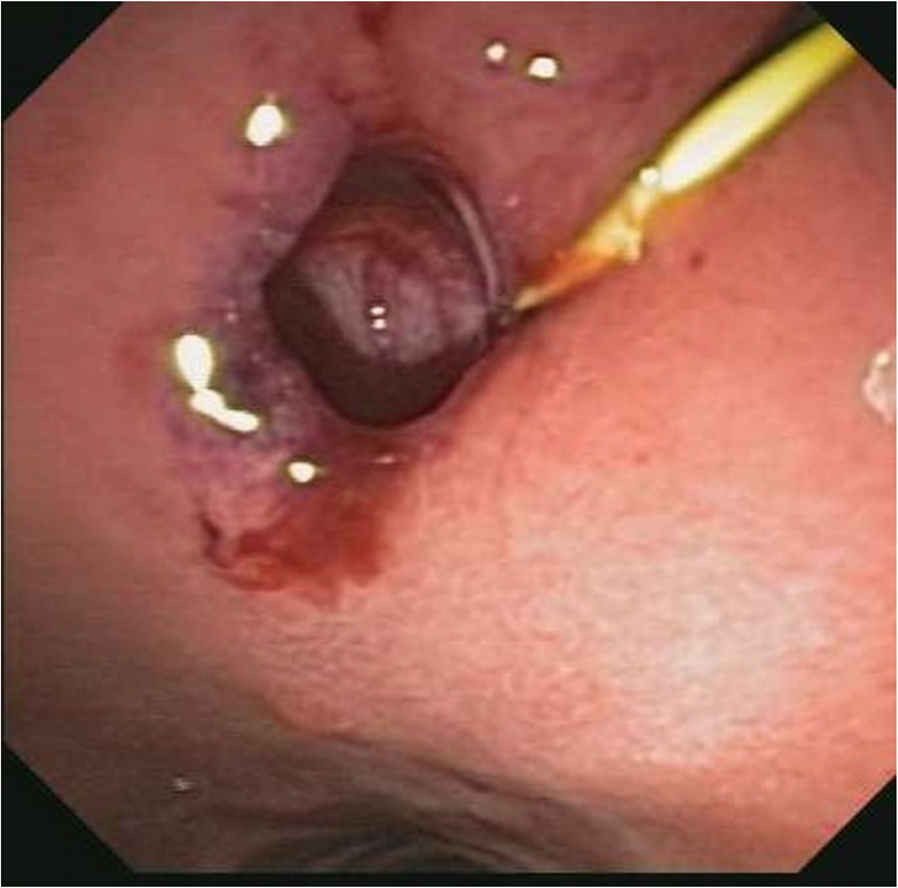
Fistula creation*

**Fig. 7:**
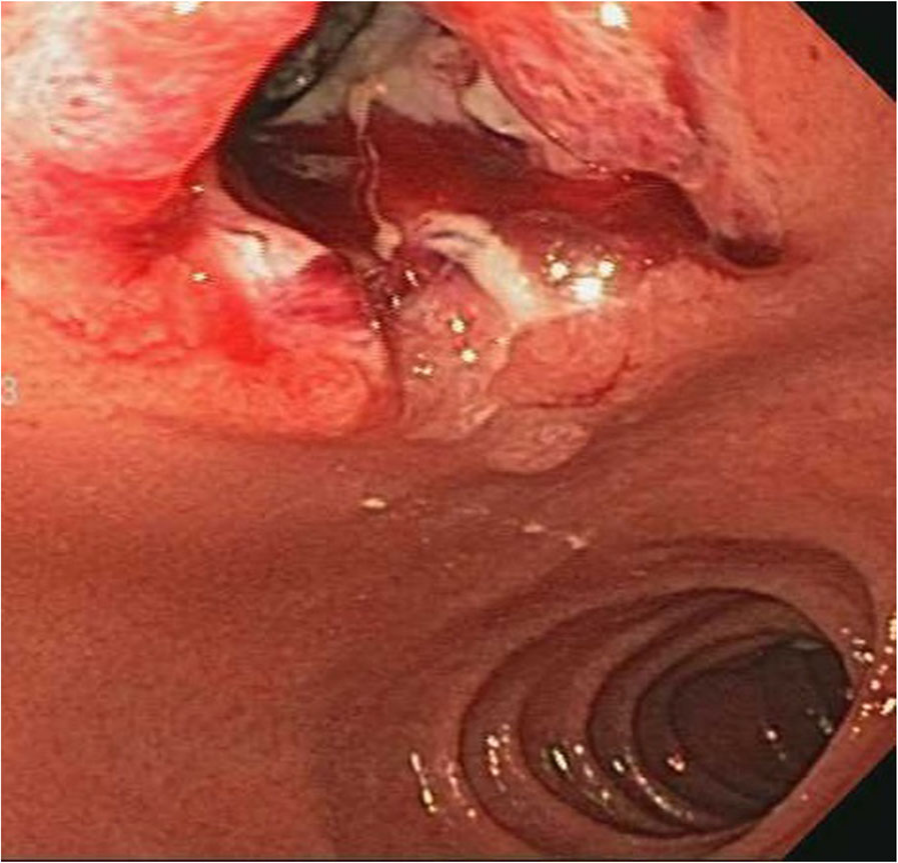
Fistula*

**Fig. 8:**
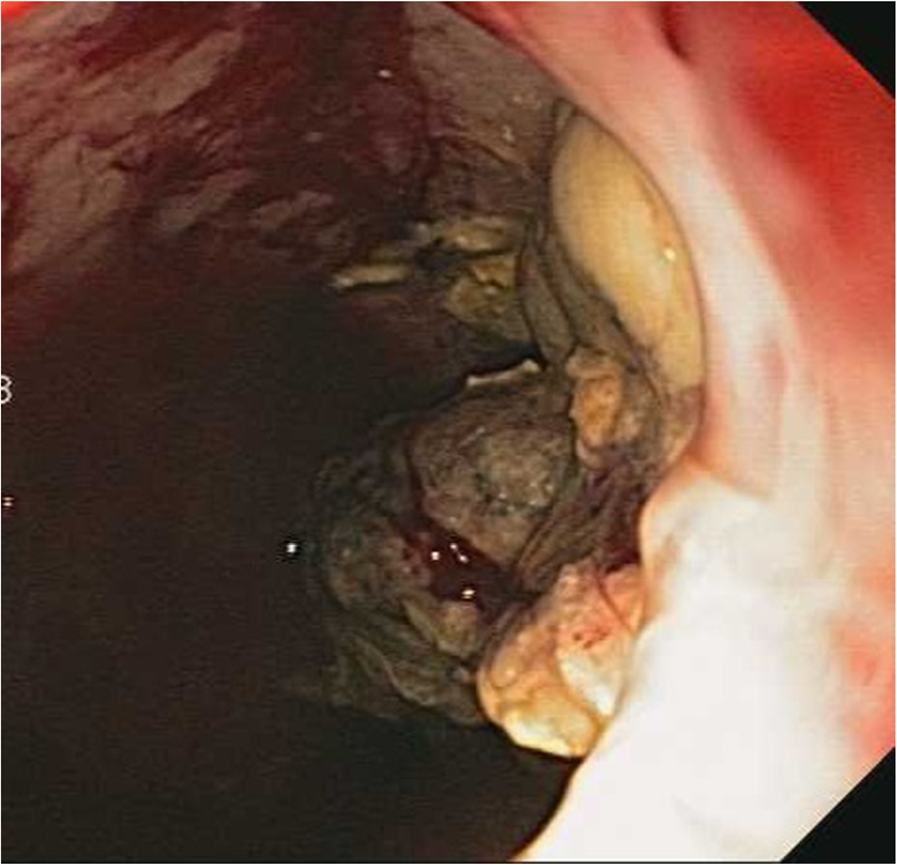
WOPN cavity*

**Fig. 9:**
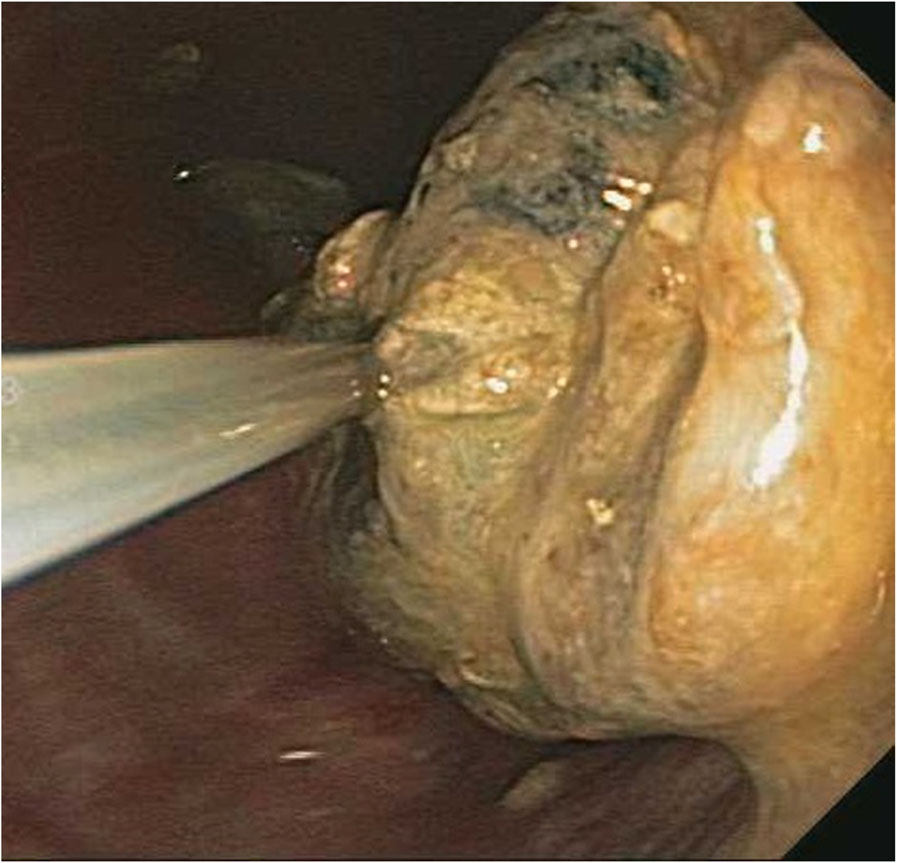
Necrosectomy

**Fig. 10:**
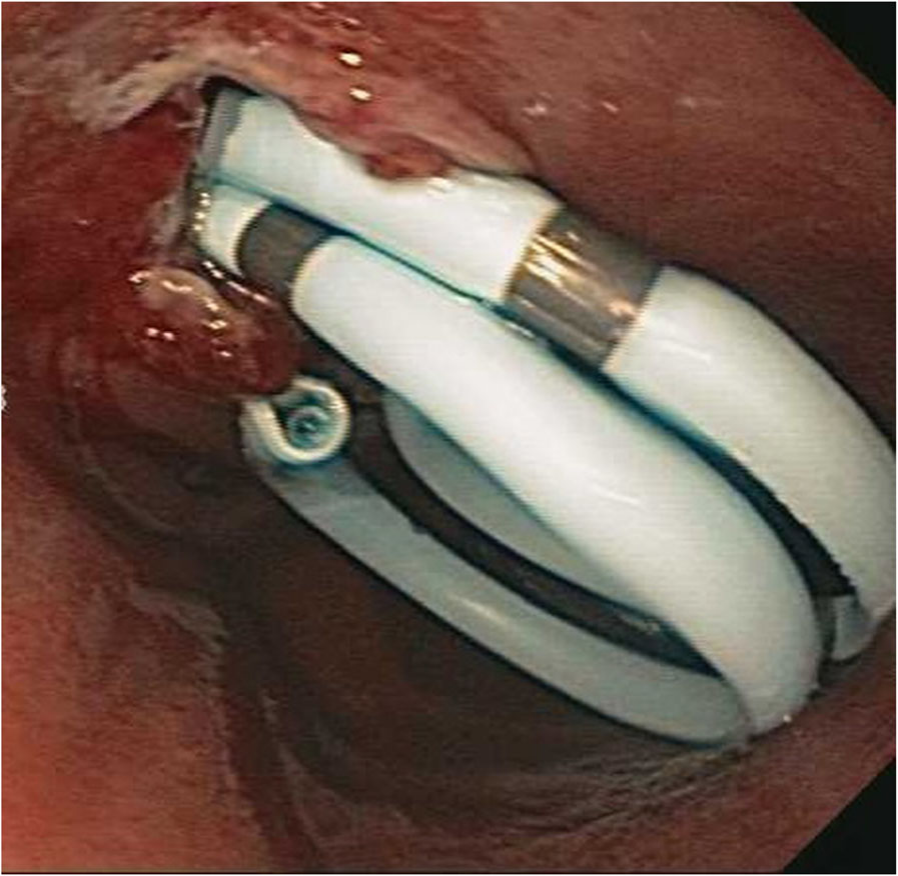
Stent placement

A 40 year old woman with a past medical history significant for morbid obesity status post sleeve gastrectomy in 2009 presented with intermittent post-prandial right upper quadrant abdominal pain consistent with symptomatic gallstone disease and was recommended for cholecystectomy. In the interim the patient developed severe necrotizing gallstone pancreatitis, which was complicated by symptomatic WOPN. At 4 week follow-up, imaging revealed a large hyperintense on T2 and hypointense on T1 fluid collection with solid components seen in the body and tail of the pancreas measuring approximately 10.8 × 7.6 cm. Follow-up imaging at week 8 showed that the WOPN was compressing the distal stomach and duodenum with symptoms of gastric outlet obstruction (Figs. 1, 2, 3 and 4). After a multi-disciplinary meeting involving surgery and interventional radiology, endoscopic necrosectomy was recommended. Esophagogastroduodenosocpy (EGD) revealed a tubular gastric body and part of the antrum, with extrinsic compression in the antrum and duodenal bulb from the WOPN (Fig. 5). The preferred fistula site was in the duodenal bulb due to distance from sleeve, cyst location and desire to avoid the staple line (Figs. 6 and 7). The patient underwent successful TDN (Figs. 8 and 9) with placement of three 10 Fr pigtail stents (Fig. 10). At 3 month follow-up, patient did well with resolution of WOPN and removal of pigtail stents.

## Conclusion

To our knowledge, this is the first reported case of a patient with sleeve gastrectomy undergoing successful endoscopic pancreatic cyst drainage and necrosectomy. Although the transgastric approach is preferred for walled-off pancreatic fluid collections due to location of the collection being more favorable and the comfort level of endoscopists, there is no definite evidence that transgastric drainage is superior to transduodenal drainage [13]. Long-term patency of transduodenal drainage tracts may reduce the recurrence rate of pancreatic pseudocysts. After the creation of a sleeve, there is increased intraluminal pressure, which incidentally is also thought to play a role in leaks along the staple line [14–17]. Specifically, a study measured the volume and pressure for both the resected stomach and the remaining sleeve and demonstrated the distensibility of the resected portion is 10-fold higher than the gastric sleeve with a significantly lower intraluminal pressure [18, 19]. They were able to conclude that the mechanism of restriction following the sleeve gastrectomy is the combination of the small capacity, low distensibility, and the resultant immediate high intraluminal pressure [19]. The duodenal approach was used in our patient to avoid the high pressure in the sleeve stomach that may lead to inadequate drainage, as fluid is known to preferentially flow from a high-pressure area to a low-pressure area. Conversely, recent studies have shown success with drainage of peritoneal fluid collections from sleeve gastrectomy leaks using endoscopically placed pigtail stents, similar to the procedure used for drainage of symptomatic pancreatic fluid collections [20–22]. Although this method has been successful for leaks, this may be aided by the leak-induced disruption of the increased intraluminal pressure and therefore may not be generalizable to draining PFCs into the sleeve. Additionally, manipulation nearby the staple line may lead to an anastomotic leak and disruption of the staple line and should be avoided. Another potential location of drainage of PFCs in this patient population is the preserved portion of the antrum. The duodenal bulb was chosen over the antrum in our patient due to the cyst location and desire to maintain distance from the staple line.

Review of literature revealed one similar case with a failed attempt at endoscopic cyst-gastrostomy due to limited working space and the inability to visualize the posterior aspect of the stomach given sleeve gastrectomy [12]. Although the surgical approach is an adequate intervention for these patients, randomized trials have validated similar outcomes between endoscopic and surgical pancreatic pseudocyst drainage, with endoscopic treatment linked with reduced hospital stays, improved physical and mental health of patients, and decreased cost [6, 23]. This case illustrates the feasibility of endoscopic drainage and necrosectomy for WOPN in patients with altered gastric anatomy through EUS-guided TDN [24].

CARE guidelines were adhered to in the publication of this manuscript [25].

## Abbreviations

EUS: Endoscopic ultrasound
WOPN: Walled off pancreatic necrosis
PFC: Pancreatic fluid collection
TDN: Transduodenal necrosectomy
EGD: Esophagogastroduodenosocpy
CT: Computed tomography
Fr: French
